# Safety of blood reinfusion drains after local infiltration analgesia in total joint replacement

**DOI:** 10.1186/s12891-024-07261-z

**Published:** 2024-02-23

**Authors:** Claudio Legnani, Enrica Torretta, Marco Attanasio, Cecilia Gelfi, Franco Parente, Alberto Ventura, Giorgio Oriani

**Affiliations:** 1 IRCCS Istituto Ortopedico Galeazzi, Sport Traumatology and Minimally Invasive Surgery Center, Milan, Italy; 2 IRCCS Istituto Ortopedico Galeazzi, Laboratory of Proteomics and Lipidomics, Milan, Italy; 3 IRCCS Istituto Ortopedico Galeazzi, Hip and Knee Arthroplasty Surgery Center, Milan, Italy; 4 Department of Biomedical Sciences for Health, University of Milan, Milan, Italy; 5 IRCCS Istituto Ortopedico Galeazzi, Department of Anesthesiology, Milan, Italy

**Keywords:** Local infiltration analgesia, Reinfusion drain, Total knee arthroplasty, Total hip arthroplasty, Levobupivacaine

## Abstract

**Background:**

Local infiltration analgesia (LIA) is frequently administered to patient undergoing joint replacement surgical procedures. The aim of the present research was to verify the safety of collected shed blood to be reinfused postoperatively, by measuring levobupivacaine levels in drainage blood in patients undergoing LIA during knee replacement surgery.

**Patients and Methods:**

24 patients who underwent total knee arthroplasty (TKA) and 12 scheduled for total hip arthroplasty (THA) who received intraoperative LIA were considered. Blood samples were collected from shed blood which was present in drainage 2 and 5 hours after surgery and serum was analysed by liquid chromatography-tandem mass spectrometry.

**Results:**

At 2 hours postoperatively, the median levobupivacaine serum concentration in the collected shed blood was 1.2 mg/L (SD: 4.2) for TKA and 17.13 mg/L (SD: 24.4) for THA. At 5 hours, levobupivacaine concentration was 1.84 mg/L (SD: 2.2) for TKA and 17.5 mg/L (SD: 25.2) for THA. Higher values of average serum levobupivacaine concentration were reported in drains collected from patients who had undergone THA compared to TKA (*p*<0.001). BMI significantly influenced levels of serum drug, that resulted to be higher in patients with BMI<25 (*p*= 0.01).

**Conclusion:**

Levobupivacaine from collected shed blood that would have been returned to the patient, was below toxicity level at 2 and 5 hours after LIA during total joint replacement. The average serum levobupivacaine concentration was found to be higher in drains taken from THA patients than TKA patients. Patients with lower BMI demonstrated the highest levels of levobupivacaine in shed blood and a lower blood volume needed for central nervous system toxicity. Therefore, in patients with a lower BMI undergoing THA, anaesthetic dosage should be reduced or autotransfusion should be avoided to prevent potential risks of toxicity.

**Supplementary Information:**

The online version contains supplementary material available at 10.1186/s12891-024-07261-z.

## Introduction

Joint replacement surgery provides satisfactory patient reported outcome scores and good to excellent long-term outcomes, although it is associated with blood loss and pain in the perioperative period [1, 2]. In order to reduce postoperative pain and to effectively control blood loss, local infiltration analgesia (LIA), tranexamic and dexamethasone injections are frequently administered to patient undergoing total joint replacement [3–5].

In presence of increased risks of bleeding, intraoperative retrieval and reinfusion of autologous blood is a common blood management technique, which reduces the drawbacks associated with allogenic transfusion [6]. However, since local anaesthetics may be present in significant concentrations in the drainage blood after joint replacement surgery, the combined use of LIA and autologous blood reinfusion is debated, as it poses inherent risks of exposure to toxic concentrations of anaesthetics, leading to potential central nervous or cardiovascular system toxicity [7].

For these reasons, determination of anaesthetics is necessary to allow a safe application of post-operative reinfusion in patients undergoing major joint replacement surgery [8].

The aim of the present research was to verify the safety of collected shed blood to be reinfused postoperatively, by measuring levobupivacaine levels in drainage blood. The hypothesis was that the concentration of anaesthetics was below toxicity threshold, thus allowing safe blood reinfusion in patients undergoing LIA during knee replacement surgery.

## Patients and methods

### Patients recruitment

Thirty-seven consecutive patients undergoing primary knee or hip arthroplasty for end-stage osteoarthritis were enrolled in the present study. Exclusion criteria were: revision surgery, septic and rheumatic arthritis, psychiatric disorders that could have compromised sample collection, contraindications to the use of LIA. Patients’ demographic and anthropometric data including surgical procedure, age at surgery, sex, body mass index (BMI) were recorded. Ethical approval was obtained. The research was conducted according to the 1964 Declaration of Helsinki and its later amendments. All patients signed written informed consent.

### Surgical technique and rehabilitation protocol

Spinal anesthesia was administered to all patients using 0.25% levobupivacaine (Chirocaine 0,625 mg/ml, AbbVie Srl, Campoverde di Aprilia, Italy). TKA was performed with a medial parapatellar approach, with the use of a tourniquet. A cemented Persona (Zimmer-Biomet Orthopedics, Warsaw, IN, USA) was implanted without performing patellar resurfacing with standard instrumentary guides. THA was performed through a posterior MIPSA (mini-invasive pyriform sparing approach) using G7 acetabular cup and Avenir uncemented stem (Zimmer-Biomet Orthopedics).

During surgery, 100 mL of a solution, containing 200 mg levobupivacaine, 8 mg dexamethasone and 1 mg adrenaline, was injected in the pericapsular soft tissue. For TKA, the posterior capsule and deep tissues were injected before prosthesis implantation, while the anterior synovium, capsule, and subcutaneous tissues were injected at the end of the surgical procedure. In THA, half of the solution was infiltrated in pericapsular soft tissues after definitive implant setting, while the remaining half was infiltrated more superficially in the musculotendinous and subcutaneous tissues.

A suction drain was placed in the joint space before capsular closure. The wound was sutured and covered with a sterile dressing. Low-vacuum suction (60 mmHg) was started after 20 min from skin closure to allow LIA to remain in the joint cavity in the early postoperative period.

A rehabilitation protocol including joint motion exercises, and progressive weight-bearing ambulation with crutches was started the day after surgery for the first 4 weeks.

### Sample collection

Blood samples (5 mL) were collected using tubes with separator gel from the suction drainage bag at 2 and 5 h after skin closure, to determine the quantity of levobupivacaine present. The time point of 5 h was chosen since it is the maximum suggested time for blood collection to allow autologous reinfusion.

All blood samples were centrifuged at 3000 revolutions per minute for 10 min to allow the removal of precipitated material and the detection of the supernatant fraction. The serum (1.5 to 2 mL) was then stored frozen at -80 °C.

### Sample preparation

50 µL of internal standard ropivacaine 0.05 ug/mL was added to 50 µL of blank, standard or patient samples and mixed together with 200 µL of methanol:acetonitrile/1:4 to precipitate the proteins. After centrifugation at 15000xg for 5 min, surnatant was diluted 1:100 in methanol:acetonitrile/1:4 and transferred in glass vials. Calibration curve standard was obtained by mixing and diluting a stock solution of 1 mg/mL of ropivacaine in methanol, covering a concentration range from 3 ng/L to 500 µg/L.

### Liquid chromatography tandem-mass spectrometry method

A volume of 5 µL of surnatant was injected into a Waters Acquity UPLC system linked to a Xevo TQ-S micro mass spectrometer (Waters, Milford, MA, USA). Ropivacaine and bupivacaine were separated on a C18 Acquity UPLC BEH 100 mm × 2.1 mm id, 1.7 μm (Waters) kept at room temperature, with the following gradient: 0.0 min: 0% B; 0.5 min: 0% B; 5.5 min: 95% B; 6.5 min: 95% B; 7 min: 0% B. The total analysis time was 9.5 min and the flow rate was set at 0.3 mL/min. Phase A consisted of 0.1% formic acid in water, while phase B was 0.1% formic acid in acetonitrile. An electrospray interface operating in positive ion mode was employed.

Data acquisition was performed via multiple reaction monitoring (MRM). Precursor to product ion transitions were 274.98 > 125.8 for ropivacaine and 289 > 140 for bupivacaine, cone voltage 28 V, collision energy 20 eV. The capillary voltage was set at 3.5 kV. The source temperature was set to 150 °C. The desolvation gas flow was set to 1000 L/hr, and the desolvation temperature was set to 500 °C. Data were acquired by MassLynx ™ 4.2 software and quantified by TargetLynx software.

### Statistical analysis

Data were analysed using Graphpad Prism v8.0 (Prism software, La Jolla, USA). Differences < 0.05 were considered statistically significant. The Shapiro-Wilk test was used to assess normal distribution of data. On the basis of these results, the comparison between groups was performed using unpaired Student t-test or Mann-Whitney test. Samples that have levels of local anaesthetic below the limit of detection (< 3 ng/L) were indicated as 0 mg/L. Cardiovascular toxicity was calculated following the formula used by Angerame MR et al. [9], (2 + 5 h mg/L)^−1^ * 1.1 mg/kg * weight in kg) that consider the lowest reported cardiovascular toxic threshold for intravenous bupivacaine (1.1 mg/kg) [10]. Central nervous system (CNS) toxicity threshold was calculated considering the lowest reported minimum bupivacaine threshold for CNS toxicity (0.3 mg/L) [11] multiplied for estimated blood volume (EBV), calculated using Nadler’s formula [12]. Blood volume needed for CNS toxicity was calculated by dividing the minimum toxic threshold to the media of the 2-hour and 5-hour drug concentrations. Before recruiting patients, a power analysis was conducted, assuming that 5% of cases undergoing LIA would show cardiac and CNS toxic values. For determining the sample size, a 7.5% absolute precision to assess the prevalence of toxicity and 95% confidence interval were used. A sample size of 33 patients was identified, which was incremented to 37 patients to allow a 10% drop out.

## Results

Patients’ demographic and anthropometric data are reported in Table 1. No significant complications, nor adverse events related to drain management were reported in any of the 37 patients recruited up to five hours after surgery.

**Table 1:** Patient Demographics and Anthropometric Data

	Overall	TKA	THA
No. of patients	37	24	13
Sex			
Male	11	6	5
Female	26	18	8
Mean BMI (SD)	28.3 (4.1)	29.6 (4.2)	26.0 (2.8)
Mean height (SD) (cm)	163.4 (8.5)	162.9 (8.5)	164.2 (8.7)
Mean age at surgery (SD) (yr)	70.9 (9.7)	70.8 (9.0)	71 (11.2)

*TKA *Total knee arthroplasty, *THA *Total hip arthroplasty, *BMI *Body Mass Index

SD Standard deviation

In patients who underwent TKA, median anaesthetic concentration was 1.2 mg/L (SD: 4.2; range, 0 to 17.5 mg/L) 2 h after skin closure, and 1.84 mg/L (SD: 2.2; range, 0 to 7.39 mg) 5 h after surgery. In THA patients, median anaesthetic concentration was 17.13 mg/L (SD: 24.4; range, 1.88 to 78.33 mg/L) after 2 h, and 17.5 mg/L (SD: 25.2; range, 0.96 to 80.7 mg/L) after 5 h. The median value of the 2-hour and 5-hour levobupivacaine concentrations was 4.2, SD: 18 (median 2.28, SD: 3.0 for TKA and median 17.24, SD: 24.8 for THA, respectively (Table 2). The autotransfusion canister output was insufficient for 1 patient at 2 h postoperatively and for 1 patient at 2 and 5 h postoperatively to allow blood samples collection.

**Table 2:** Results of the drain output levobupivacaine concentration

	Median levobupivacaine concentration (mg/L)		
	2 h (SD)	5 h (SD)	2 h + 5 h (SD)
Overall	4.4 (18.4)	3.6 (18.1)	4.2 (18)
TKA	1.20 (4.2)	1.84 (2.2)	2.28 (3.0)
THA	17.13 (24.4)	17.5 (25.2)	17.24 (24.8)

*TKA *Total knee arthroplasty, *THA *Total hip arthroplasty, *SD *Standard deviation

**Fig. 1:**
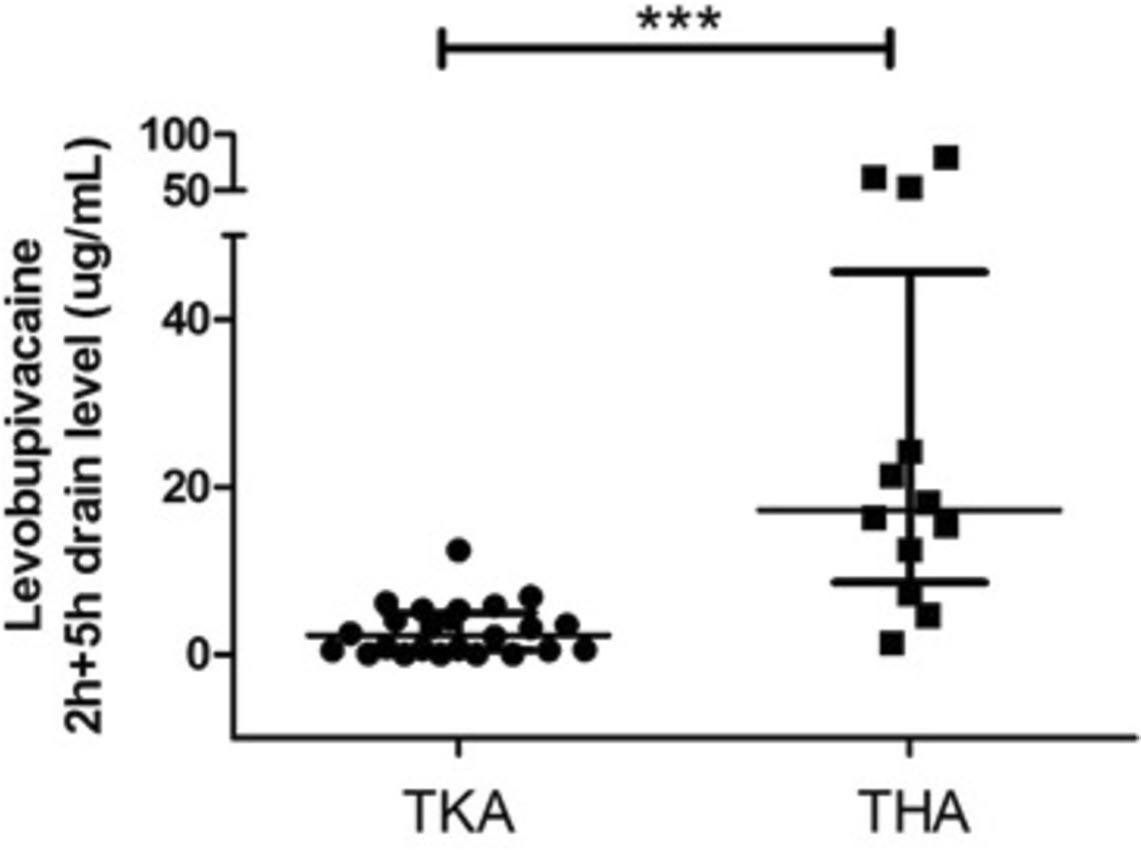
Scatter plot showing levels of levobupivacaine (mg/L) in patients who underwent TKA and THA.  *** Mann Whitney test *p*-value < 0.001

 Details of the drain output levobupivacaine concentration at the settled time points are reported in Appendix: Table 3. Higher values of average serum levobupivacaine concentration were reported in drains collected from patients who had undergone THA compared to TKA (*p* < 0.001; Fig. 1).

**Fig. 2:**
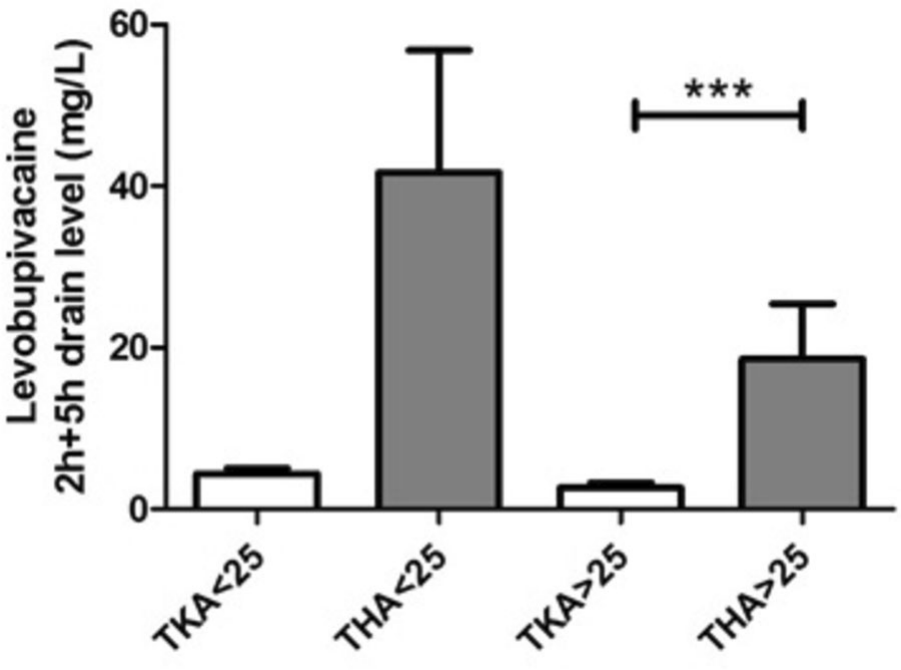
Levels of levobupivacaine (mean ± SEM) in patients who underwent TKA (white bars) and THA (grey bars), subgrouped according to BMI (< 25 or > 25).  *** Mann Whitney test *p*-value < 0.001

Patients who underwent TKA and THA were divided into two groups based on their BMI; BMI > 25 was used as a cut-off point for classifying overweight. Mean levobupivacaine levels resulted to be higher in patients with a BMI < 25 (*p* = 0.01). Patients who underwent THA were characterized by a lower average BMI compared to TKA (*p* = 0.01), however they showed higher levels of drug even when subgrouped according to BMI (Fig. 2).

**Fig. 3:**
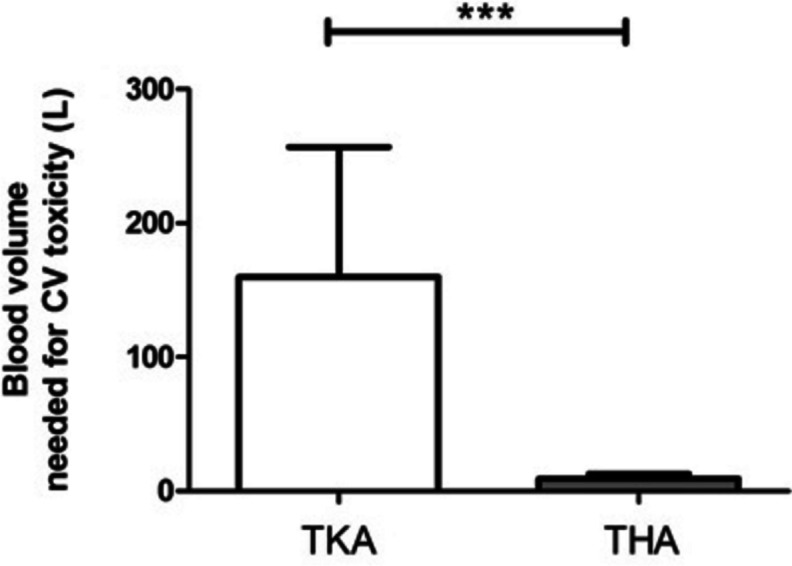
Blood volume needed to trigger cardiovascular (CV) toxicity in patients who underwent TKA (white bar) and THA (grey bar).  *** Mann Whitney test *p*-value < 0.001

 The minimum cardiotoxic level for each patient was computed based on each person’s weight and the lowest toxic intravenous bupivacaine level documented in the literature (1.1 mg/kg) [10]. The average blood volume needed to reach cardiotoxicity was calculated to be greater than 159 L in TKA operated patients and over 9 L in patients who underwent THA (*p* < 0.001, Appendix: Table 4; Fig. 3).

**Fig. 4:**
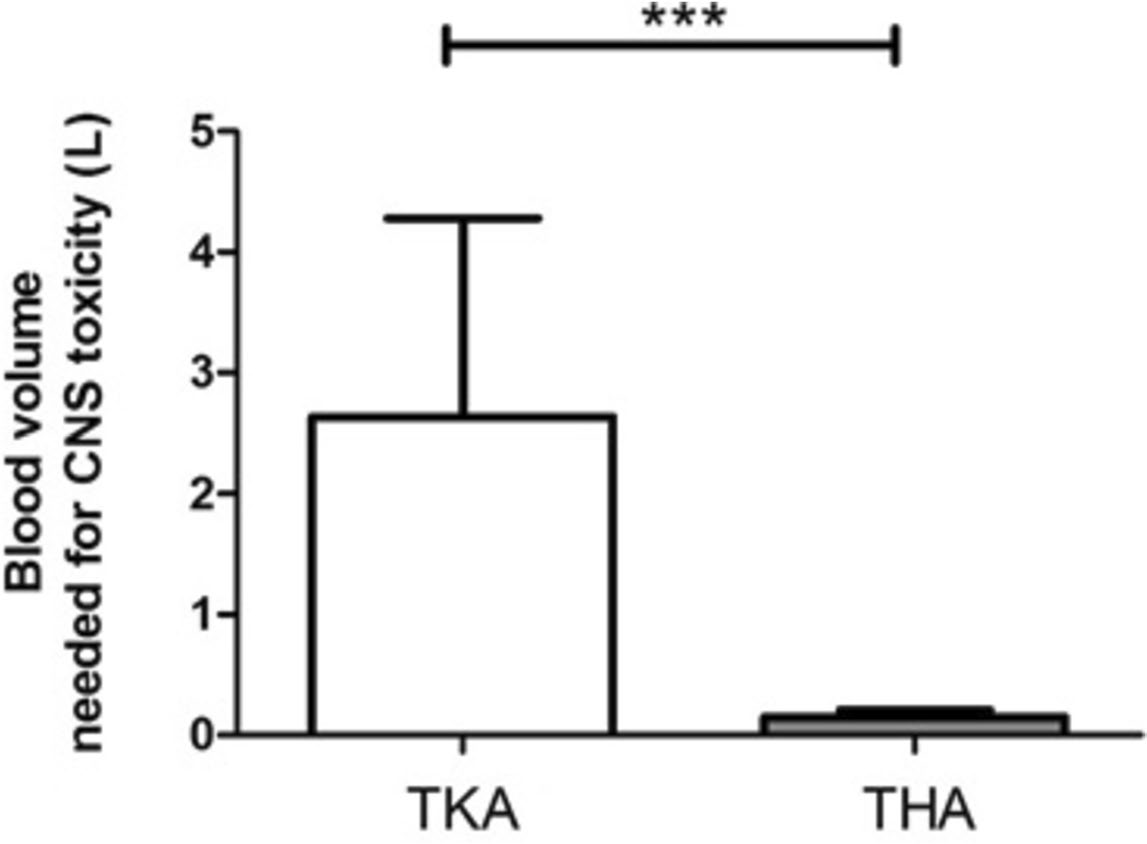
Blood volume needed to trigger central nervous system (CNS) toxicity in patients who underwent TKA (white bar) and THA (grey bar).  *** Mann Whitney test *p*-value < 0.001

 Based on the lowest threshold reported (0.3 mg/L) [11] and using EBV, individual minimum bupivacaine level thresholds for CNS toxicity were assessed. Mean levobupivacaine thresholds for CNS toxicity were 1.33 mg (SD: 0.25) and 1.28 mg (SD: 0.16) for TKA and THA, respectively. The average volume of autotransfused blood required to cause CNS toxicity was 2.63 L in TKA group and 0.15 L for THAs (*p* < 0.001, Fig. 4). Higher levels of drug concentrations in patients that underwent THA lowered the amount of blood volume needed to reach CNS toxicity, as shown in Appendix: Table 5.

## Discussion

The results of the present study showed that the amount of levobupivacaine in the autotransfused blood was below the minimal toxic serum dose that has been documented.

The average serum levobupivacaine concentration was found to be higher in drains taken from THA patients than TKA patients. In addition, patients with lower BMI demonstrated the highest levels of levobupivacaine in shed blood and a lower blood volume needed for CNS toxicity.

LIA is recognized as an effective measure to provide pain reduction and allow faster rehabilitation and reduced hospital stay following joint replacement [3, 6]. Levobupivacaine is frequently used as anaesthetic in LIA and demonstrated satisfying outcome in patients undergoing TKA and THA [13, 14].

At present, few publications with a low number of patients enrolled reported on the safety of autologous blood transfusion following levobupivacaine local injection [9, 15, 16].

Wallace et al. quantified levobupivacaine level in blood and drain samples of 23 patients who underwent total knee arthroplasty in combination with LIA. An average level of 4.9 µg/ml (SD 3.1) was identified in the drain fluid. The analysis of blood samples following blood reinfusion did not demonstrated toxic quantities of anaesthetic in the circulatory system [15, 16].

Similarly to our study, Angerame et al. analysed bupivacaine levels in blood samples collected from drain at 2 and 5 h following joint arthroplasty in 11 patients. Average level at 2 h was  2.9 µg, while mean level was 4.35 µg at 5-hour timepoint. Authors conclude that values were well below toxicity threshold and therefore autotransfusion should be safe [9].

Our findings corroborate previous investigations that have shown that autotransfusion from reinfusion drains is secure following LIA in patients undergoing total joint replacement.

In our case series, the levobupivacaine toxicity threshold for the cardiovascular system was not reached in any of the research participants. The difference was less significant for CNS toxicity thresholds and drain serum levobupivacaine levels, with patients with lower BMI demonstrating a lower blood volume needed for CNS toxicity. According to our findings, depending on the patient’s height and weight, 0.09 to 33.6 L of blood would need to be transfused in order for the concentrations of levobupivacaine to exceed the lowest known CNS hazardous threshold of 300 mg/L following TKA. Conversely, following THA, a minimum value of 0.015 L has been reported, thus demonstrating a lower amount of blood to be transfused to reach toxic values of anaesthetics.

According to our results, regarding TKA, at all time points, average serum concentration was found to be well below the minimum threshold for toxicity, which has been reported to range between 0.24 and 0.3 mg/L [11, 17].

Conversely, statistically significant higher values of average serum levobupivacaine concentration were reported in drains collected from patients who had undergone THA compared to TKA.

This may be explained by a presumably higher absorption from periarticular tissues in the knee, given the anatomy of the capsular structures allowing less injected drugs to be released in the surrounding tissues compared to the hip joint during pericapsular infiltration, allowing prolonged intraarticular drug persistence. Another additional reason may be the fact that the compression bandage used in the knee following TKA has the potential to prolong the permanence of anaesthetics in the injected tissues retarding its washout, thus reducing systemic absorption [17].

More interestingly, our study is the first to demonstrate that levobupivacaine level in shed blood may approach toxic level in some conditions. BMI significantly influenced levels of serum drug, that resulted to be higher in patients with a BMI < 25. Specifically, patients undergoing THA with lower BMI demonstrated the highest levels of levobupivacaine in collected blood and a lower blood volume needed for cardiac and CNS toxicity.

These findings may suggest to adjust clinical practice in presence of patients with lower BMI undergoing THA. In these patients, anaesthetic dosage should be reduced without significant impact on postoperative course given the fact that THA is associated lower pain compared to TKA; or physician could consider to avoid reinfusion since blood loss may be relatively limited in presence of tranexamic and adrenaline local injection.

The present study possesses limitations. It is a single-centre study with a relatively small sample size. To recruit a larger number of patients, categorizing them by both BMI, and considering TKA and THA separately could add statistical validity to these findings, enhancing the solidity of the results. In addition, no information about patients’ venous blood levels of levobupivacaine could be retrieved since the collected blood shed is not routinely infused at our Institution in patients undergoing LIA during elective total joint arthroplasty surgery. A baseline difference was present in BMI values between patients who underwent TKA and THA, however higher levels of levobupivacaine in THA were associated to the surgical site.

However, given these limitations, the findings of the present prospective observational study have the potential to provide information about the safety of reinfusion of filtered autologous blood during the first 5 h postoperatively when LIA has been performed after major orthopaedics surgical interventions. Further studies are required to support the current findings.

## Conclusions

Levobupivacaine from collected shed blood that would have been returned to the patient, was below the toxicity level at 2 and 5 h after LIA during total joint replacement. Higher values of average serum levobupivacaine concentration were reported in drains collected from patients who had undergone THA compared to TKA and in patients with BMI < 25 compared to BMI > 25. Patients undergoing THA required a lower blood volume to be transfused in order to reach cardiac and CNS toxicity. Therefore, according to our results, in patients with a lower BMI undergoing THA, anaesthetic dosage should be reduced or autotransfusion should be avoided to prevent potential risks of toxicity.

### Supplementary Information

**Supplementary Material 1.**

## Data Availability

The datasets used and/or analysed during the current study are available from the corresponding author on reasonable request.
