# Supplementary material for: Safety of blood reinfusion drains after local infiltration analgesia in total joint replacement
Source: BMC Musculoskelet Disord. 2024 Feb 23;25:170. doi: 10.1186/s12891-024-07261-z (PMC10885553; doi:10.1186/s12891-024-07261-z)
Supplement: Supplementary file 1 — Supplementary Material 1. [file 12891_2024_7261_MOESM1_ESM.doc]

**Table 3**

Details of the drain output levobupivacaine concentration

|  |  |  |  | Levobupivacaine concentration (mg/L) | | |
| --- | --- | --- | --- | --- | --- | --- |
| Patient no. | Sex | Surgery | BMI | 2h | 5h | 2h + 5h |
| Patient 1 | F | TKA | 31.2 | 0.44 | 0.76 | 0.60 |
| Patient 2 | F | TKA | 31.3 | 4.49 | 3.93 | 4.21 |
| Patient 3 | M | TKA | 29.4 | 0.00 | 0.00 | 0.00 |
| Patient 4 | M | TKA | 27.4 | 0.04 | 0.04 | 0.04 |
| Patient 5 | F | THA | 25.2 | 1.88 | 0.96 | 1.42 |
| Patient 6 | F | THA | 32.4 | 4.96 | 4.36 | 4.66 |
| Patient 7 | F | TKA | 25.0 | 17.50 | 7.39 | 12.44 |
| Patient 8 | M | THA | 28.0 | *0.00 | *0.00 | *0.00 |
| Patient 9 | F | TKA | 32.0 | 4.70 | 3.49 | 4.10 |
| Patient 10 | F | TKA | 36.2 | 0.00 | 0.00 | 0.00 |
| Patient 11 | F | TKA | 28.4 | 0.99 | 0.27 | 0.63 |
| Patient 12 | F | TKA | 27.8 | 0.00 | 0.00 | 0.00 |
| Patient 13 | F | TKA | 27.5 | 0.56 | 0.42 | 0.49 |
| Patient 14 | F | TKA | 27.5 | 1.20 | 0.42 | 0.81 |
| Patient 15 | F | TKA | 27.3 | 0.00 | 0.00 | 0.00 |
| Patient 16 | F | TKA | 23.8 | 0.00 | 5.74 | 5.74 |
| Patient 17 | M | THA | 24.2 | 78.33 | 80.70 | 79.51 |
| Patient 18 | M | TKA | 27.2 | *0.00 | 5.30 | 5.30 |
| Patient 19 | F | TKA | 41.9 | 2.92 | 1.27 | 2.09 |
| Patient 20 | F | TKA | 32.0 | 8.64 | 3.61 | 6.12 |
| Patient 21 | F | THA | 26.5 | 16.43 | 8.64 | 12.53 |
| Patient 22 | F | THA | 25.2 | 8.91 | 5.60 | 7.26 |
| Patient 23 | M | TKA | 23.8 | 4.44 | 2.55 | 3.49 |
| Patient 24 | F | THA | 25.3 | 62.97 | 61.02 | 61.99 |
| Patient 25 | F | THA | 24.8 | 53.58 | 51.90 | 52.74 |
| Patient 26 | M | TKA | 25.8 | 9.49 | 4.23 | 6.86 |
| Patient 27 | M | TKA | 33.2 | 5.24 | 5.27 | 5.25 |
| Patient 28 | F | THA | 24.0 | 16.11 | 16.51 | 16.31 |
| Patient 29 | F | THA | 27.9 | 22.22 | 20.57 | 21.40 |
| Patient 30 | F | THA | 20.6 | 17.84 | 18.49 | 18.17 |
| Patient 31 | F | THA | 27.7 | 28.40 | 20.24 | 24.32 |
| Patient 32 | M | THA | 26.7 | 15.02 | 15.78 | 15.40 |
| Patient 33 | F | TKA | 34.1 | 3.37 | 2.96 | 3.16 |
| Patient 34 | F | TKA | 34.1 | 3.47 | 2.75 | 3.11 |
| Patient 35 | F | TKA | 27.3 | 2.52 | 2.41 | 2.47 |
| Patient 36 | M | TKA | 29.4 | 0.50 | 0.51 | 0.51 |
| Patient 37 | M | TKA | 26.4 | 0.52 | 0.52 | 0.52 |

TKA: Total knee arthroplasty THA: Total hip arthroplasty

* insufficient sample

**Table 4**

Blood volume needed for cardiovascular toxicity according to weight

| Patient no. | Weight (Kg) | Blood volume needed for cardiovascular toxicity (L) |
| --- | --- | --- |
| Patient 1 | 78 | 143.0 |
| Patient 2 | 80 | 20.9 |
| Patient 3 | 78 | ND |
| Patient 4 | 72 | 1980.0 |
| Patient 5 | 59 | 45.7 |
| Patient 6 | 105 | 24.8 |
| Patient 7 | 64 | 5.7 |
| Patient 8 | 81 | ND |
| Patient 9 | 82 | 22.0 |
| Patient 10 | 71 | ND |
| Patient 11 | 88 | 153.6 |
| Patient 12 | 65 | ND |
| Patient 13 | 74 | 166.1 |
| Patient 14 | 74 | 100.5 |
| Patient 15 | 70 | ND |
| Patient 16 | 72 | 13.8 |
| Patient 17 | 70 | 0.97 |
| Patient 18 | 74 | 15.4 |
| Patient 19 | 110 | 57.9 |
| Patient 20 | 85 | 15.3 |
| Patient 21 | 74 | 6.5 |
| Patient 22 | 62 | 9.4 |
| Patient 23 | 73 | 23.0 |
| Patient 24 | 57 | 1.0 |
| Patient 25 | 75 | 1.6 |
| Patient 26 | 78 | 12.5 |
| Patient 27 | 84 | 17.6 |
| Patient 28 | 60 | 4.0 |
| Patient 29 | 76 | 3.9 |
| Patient 30 | 52 | 3.2 |
| Patient 31 | 80 | 3.6 |
| Patient 32 | 70 | 5.0 |
| Patient 33 | 82 | 28.5 |
| Patient 34 | 82 | 29.0 |
| Patient 35 | 70 | 31.2 |
| Patient 36 | 90 | 194.0 |
| Patient 37 | 80 | 169.0 |

ND: Not detectable

**Table 5**

Blood volume needed for CNS toxicity according to Nadler’s formula

| Patient no. | EBV (L) | CNS toxicity threshold (mg) | Blood volume needed for CNS toxicity (L) |
| --- | --- | --- | --- |
| Patient 1 | 4.17 | 1.25 | 2.08 |
| Patient 2 | 4.29 | 1.29 | 0.3 |
| Patient 3 | 4.70 | 1.41 | ND |
| Patient 4 | 4.48 | 1.34 | 33.6 |
| Patient 5 | 3.41 | 1.02 | 0.72 |
| Patient 6 | 6.12 | 1.84 | 0.39 |
| Patient 7 | 3.76 | 1.13 | 0.09 |
| Patient 8 | 5.01 | 1.5 | ND |
| Patient 9 | 4.35 | 1.31 | 0.32 |
| Patient 10 | 3.51 | 1.05 | ND |
| Patient 11 | 5.44 | 1.63 | 2.59 |
| Patient 12 | 3.61 | 1.08 | ND |
| Patient 13 | 4.20 | 1.26 | 2.57 |
| Patient 14 | 4.20 | 1.26 | 1.56 |
| Patient 15 | 3.96 | 1.19 | ND |
| Patient 16 | 4.44 | 1.33 | 0.23 |
| Patient 17 | 4.25 | 1.27 | 0.02 |
| Patient 18 | 4.23 | 1.27 | 0.24 |
| Patient 19 | 5.34 | 1.6 | 0.76 |
| Patient 20 | 4.54 | 1.36 | 0.22 |
| Patient 21 | 4.29 | 1.29 | 0.1 |
| Patient 22 | 3.61 | 1.08 | 0.15 |
| Patient 23 | 4.92 | 1.48 | 0.42 |
| Patient 24 | 3.27 | 0.98 | 0.02 |
| Patient 25 | 4.95 | 1.48 | 0.03 |
| Patient 26 | 5.05 | 1.51 | 0.22 |
| Patient 27 | 4.39 | 1.32 | 0.25 |
| Patient 28 | 3.57 | 1.07 | 0.07 |
| Patient 29 | 4.30 | 1.29 | 0.06 |
| Patient 30 | 3.33 | 1.0 | 0.05 |
| Patient 31 | 4.98 | 1.49 | 0.06 |
| Patient 32 | 4.42 | 1.32 | 0.09 |
| Patient 33 | 4.22 | 1.27 | 0.4 |
| Patient 34 | 4.22 | 1.27 | 0.41 |
| Patient 35 | 3.96 | 1.19 | 0.48 |
| Patient 36 | 5.47 | 1.64 | 3.22 |
| Patient 37 | 4.71 | 1.41 | 2.71 |

EBV: estimated blood volume CNS: central nervous system ND: Not detectable
